# Development, Characterization and Pharmacokinetic Profile of Chitosan-Sodium Tripolyphosphate Nanoparticles Based Drug Delivery Systems for Curcumin

**DOI:** 10.34172/apb.2021.008

**Published:** 2020-11-07

**Authors:** Wawaimuli Arozal, Melva Louisa, Deni Rahmat, Priska Chendrana, Ni Made Dwi Sandhiutami

**Affiliations:** ^1^Department of Pharmacology and Therapeutics, Faculty of Medicine, Universitas Indonesia, Jakarta, Indonesia.; ^2^Faculty of Pharmacy, University of Pancasila, Jakarta, Indonesia.; ^3^Doctoral Program in Biomedical Sciences, Faculty of Medicine, Universitas Indonesia, Jakarta, Indonesia.

**Keywords:** Curcumin, Chitosan, Nanoparticles, Mucoadhesive, Pharmacokinetics

## Abstract

***Purpose:*** This study aimed to provide the method of preparation, characterization of curcumin-loaded chitosan-sodium tripolyphosphate (NaTPP) nanoparticle, and evaluate its pharmacokinetic profiles.

***Methods:*** Curcumin-loaded chitosan-NaTPP nanoparticles were synthesized using ionic gelation methods. Curcumin was dissolved using surfactants and cosurfactants. Chitosan polymer was then mixed in the curcumin solution and dripped with NaTPP solution until nanoparticle formation. The mucoadhesive study was evaluated by measuring the fluorescence of curcumin within the prepared nanoparticles. The pharmacokinetic profiles of curcumin particles and nanoparticles were then assessed in rats by administering a single oral dose of 100 mg/kg BW. Blood samples were taken from nine predetermined time points, and curcumin plasma concentrations were then analyzed using UPLC-MS/MS.

***Results:*** The particle size of the curcumin nanoparticles obtained were 11.5 nm. Entrapment efficiency (EE) of curcumin nanoparticles were exceeding 99.97%, and drug loading capacity (DLC) was 11.34%. The mucoadhesive properties of the nanoparticles were superior to that of curcumin particles. Pharmacokinetic evaluation in rats revealed that curcumin nanoparticles resulted in an increase of area under the curve (AUC), maximum concentration (Cmax), earlier time to reach maximum concentration (Tmax), and lower clearance (CL).

***Conclusion:*** Curcumin-loaded chitosan-NaTPP nanoparticles is an effective formulation to improve curcumin plasma concentrations. Thus, enable its applications for the treatment of various diseases.

## Introduction

Curcumin is a compound derived from a natural agent, also known as diferuloylmethane. This agent is one of the lipophilic phenolic compounds in the *Curcuma longa* plant, which is known as *Curcuma domestica* and is widely found in Southern Asia, India, Indochina, and other Asian countries. Various research has studied pharmacological effects of curcumin, including its anti-inflammatory, antioxidant, antiviral, antifungal, antibacterial, immunomodulatory, and anticancer effects.^1,2^ Curcumin exerts its anticancer effects by suppressing tumor growth, increasing apoptosis, and modulating the multistep carcinogenesis processes.^3,4^ In prostate and lung cancer line cells, curcumin reduces VEGF expressions as well as MMP-9 and MMP-2 activities. Long-term use of curcumin in liver cancer mice models was showed to inhibit angiogenesis by reducing MMP-2, MMP-9, PKC-a, and VEGF expressions. Curcumin was reported to reduce the IL-6 and VEGF in chondrosarcoma cell lines.^5,6^

Curcumin has a low bioavailability, owing to its limited absorption, extensive metabolism, and rapid elimination from the body. Low bioavailability of oral curcumin primarily due to its low water solubility and high first-pass metabolism.^7,8^ Pan et al, in their study in mice, discovered that the administration of curcumin of 1 g/kg BW, resulted in a low Cmax, and the curcumin plasma concentrations continued to decline and were undetectable after 6 hours. Also, after intraperitoneal administration of 100 mg/kg BW, curcumin concentration decreased rapidly in 1 hour.^9^ Yang et al were shown that the bioavailability of curcumin approximately 1%, with a half-life (t_1/2_) of 28.1 ± 5.6 minutes.^10^ Similarly, in humans, the plasma level of curcumin is low after 0.5 and 1 hour of oral administration at a dose of 3.6 g.^11^ Solubility in water and permeability in membranes are essential parameters in the absorption process to affect the bioavailability of the drug.

Curcumin is a hydrophobic compound that is practically insoluble in water^7^ (its solubility in water is 400 ng/mL at pH 7.4^12^) and is more soluble in organic solvents such as acetone, methanol, ethanol, and hexane. In DMSO, the solubility of curcumin at 11 mg/mL. Limitations of curcumin can be diminished by increasing its bioavailability, avoiding degradation, reducing metabolism, and increasing its loading capacity to the target organ. The challenge of preparing curcumin nanoparticle formulation is one of the innovations in increasing bioavailability, plasma concentrations, and cellular permeability, as well as inhibiting the rapid metabolic process of curcumin.^13^ A nanoparticle is a new kind of formulation that uses a nanoscale-sized particle that can increase the penetration and residence time of a drug. In its development, nanoparticles are also widely used in pharmaceutical technology as a drug delivery system. Medicines that are made in the form of nanoparticles will be absorbed in a specific place or target of the desired drug work.^14,15^

Nanoparticle formulation expected to stabilize curcumin in a physiological environment, which resulted in longer circulation time in the body and extended time in the reticuloendothelial system (RES). Further, lower clearance (CL) and a longer half-life (t_1/2_) achieved. Thus, the drug might avoid opsonization and increase endocytosis and uptake by tumor cells.^16^

There are several methods in making nanoparticles, one of which is the ionic gelation method that uses a mixture of polymers that are crossed between cationic/anionic polymers with one another on opposite ion charges. One of the polymers with excellent properties for nanoparticle formulation is chitosan. Chitosan is inert, biodegradable, biocompatible, inexpensive, and also known to have unique bioadhesive properties. Therefore, chitosan can used in promising drug delivery systems.^17,18^

The present study aimed to develop chitosan-NaTPP-based curcumin nanoparticles using the ionic gelation method, which was easy and uncomplicated. The current process does not utilize high pressure to obtain small particles. Enhanced mucoadhesive properties expected from the process. Thus, it will result in the improvement of curcumin pharmacokinetic profiles.

## Materials and Methods

Curcumin (PT. Plamed Green Science Limited, Shaanxi, China; total curcuminoid content, 95%), CMC-Na, sodium tripolyphosphate (Brataco, Bekasi, Indonesia), standard curcumin, chitosan, Kolliphor EL, glacial acetic acid, hydrochloric acid (HCl), dimethyl sulfoxide (DMSO), ethanol, propylene glycol, tween 80, glycerin were purchased from Sigma-Aldrich, St. Louis, Missouri, USA, pieces of intestinal tissue, 0.9% NaCl (PT. Widatra Bhakti, Indonesia), propranolol (internal standard), methyl tert-butyl ether/MTBE, 0.1% formic acid, acetonitrile, methanol were purchased from Merck, Darmstadt, Germany.

### Curcumin nanoparticle formulation 

Curcumin nanoparticles were made at the Faculty of Pharmacy, University of Pancasila. One gram of curcumin dissolved in mixed surfactant and cosurfactant (20 mL of propylene glycol, 10 mL of ethanol 70%, 2 mL of DMSO 10%, 4 mL of tween 80, 10 mL of glycerine, and 14 mL of Kolliphor EL). Chitosan polymer solution 1% (30 mL) was mixed in the curcumin solution using a magnetic stirrer (Thermolyne, USA) at a speed of 300 rpm at room temperature. Then, the mixture was dripped with NaTPP (10 mL) at a rate of one drop/3 second using a burette and in a 300-rpm magnetic stirrer to form nanoparticles. Afterward, the mixture remained distilled over the magnetic stirrer for 15 minutes to obtain a stable curcumin nanoparticle solution. We then observed the stability of curcumin nanoparticles for five days, including color, turbidity, and sedimentation. The characteristics of the curcumin-loaded chitosan nanoparticle, such as the average particle size and size distribution, zeta potential (ζ potential), polydispersity index (PI), entrapment efficiency (EE), and drug loading capacity (DLC), and transmission electron microscope (TEM) images were determined.

### Evaluation and characterization of curcumin nanoparticle 

#### Particle size and distribution nanoparticles

The examination of particle size and distribution of particles from nanoparticles was done using the Delsa™ Nanoparticle Size Analyzer (Beckman Coulter, Brea, USA).

#### Zeta potential measurement

We analyzed the particle charge as ζ potential using a *Zetasizer* Delsa™ Nano (Beckman Coulter, Brea, USA).

#### Particle morphology examination

TEM JEOL 1010 (80.0 kV) was used to observe the surface morphology and microscopic structure characterization of curcumin nanoparticle.

#### Determination of EE and DLC of curcumin nanoparticles

Curcumin nanoparticles having 3 mL volume were placed into centrifugation tubes, then dispersed in water to 30 mL, and centrifuged at a speed of 10 000 rpm for 30 minutes. Centrifugation results were obtained by the supernatant and sediment section. The supernatant was taken, and its absorption was measured by a 1900-UV UV-visible spectrophotometer (Shimadzu, Japan) at λ 422.5 nm. Further, absorption data was used to calculate the free curcumin weight of the formula using the linear regression equation that has been obtained from the calibration curve. After getting the weights from the sample, the EE and DLC were calculated.

Calculation of EE:

$$Entrapment\;Efficiency(EE)=\frac{C_{0}-C_{1}}{C_{0}}x100\%$$

C_0_ = the weight of the active compound at first (mg)

C_1_ = free active compound weight (mg)

Calculation of DLC:

$$Drug\;Loading\;Capacity(DLC)=\frac{C_{0}-C_{1}}{C_{total}}x100\%$$

C_0_ = the weight of the active compound at first (mg)

C_1_ = free active compound weight (mg)

C_total_ = total weight of nanoparticles (mg)

#### Released of curcumin in vitro

A volume of dissolution medium, i.e., 0.2 M phosphate buffer, pH 6.8, is put in a 500 mL container. A total of 2.5 mL of curcumin nanoparticles were inserted into the dialysis membrane placed into a medium-containing container at a speed of 50 rpm. At the same time, the stopwatch was turned on simultaneously. A 5 mL of samples were taken at 15, 30, 45, 60, 90, 120, 150, and 180 minutes in the middle area between the surface of the dissolving medium and the top of the container and not less than 1 cm from the surface of the container wall and then were dissolved in 5.0 mL of 96% ethanol. Each sample taken from the time points mentioned above was measured using a 1900-UV UV-visible spectrophotometer (Shimadzu, Japan) at λ 422.5 nm. Then, the concentration was calculated using the linear regression equation that had been obtained. Further, curcumin concentrations were calculated base on the amount of curcumin released from the matrix during testing.

#### The mucoadhesive characteristics of curcumin nanoparticles

In brief, fresh porcine intestinal mucosa was mounted on the peristaltic pipe (Drifton, Denmark) and placed at an angle of 45° in an incubation chamber, providing 100% humidity and a temperature of 37°C. The intestinal mucosa was humidified for an equilibration period of 5 minutes. Furthermore, 40 µL of curcumin nanoparticles were administered into the intestine mucosa and left for 1 hour. Afterward, curcumin nanoparticles in the intestine were rinsed with phosphate buffer, pH 6.8, at 37°C, at a constant rate of 1 mL/min. Afterward, the membrane was incubated in 30 mL of 5M NaOH for 20 minutes at 37°C. Following centrifugation (13400 rpm; 5 min), the fluorescence of each sample was evaluated using a spectrofluorometer (Hitachi F-2700) at Em/Ex = 522.5/413.5.^19-21^

### In vivo pharmacokinetic study

The pharmacokinetic evaluation was carried out after obtaining approval from the Ethics Committee of the Faculty of Medicine Universitas Indonesia (approval number: 1188/ UN.2 F1/ETIK/2018). A pharmacokinetic study was carried out in female Wistar rats to determine the pharmacokinetic profile of curcumin particles and nanoparticles. Rats weighing 150–200 g were obtained and maintained at the Biomedical Research Center and Basic Health Technology Laboratory, Balitbangkes, Republic of Indonesia, Ministry of Health. The rats were placed in a room with constant temperature and humidity, adequate lighting, pellet food, and water *ad libitum.*

A total of 60 rats were randomly divided into two groups to receive curcumin particles or curcumin nanoparticles at a dose of 100 mg/kg BW orally, five measurements for each group at each time point. For each analysis, blood samples were pooled from six rats. Curcumin nanoparticles were administered to the rats using its final formulation, while curcumin particles in CMC-Na 0.5% suspension. After administration of the sample, each of the rats from the curcumin particle group and the curcumin nanoparticles group was taken for blood at 0, 1, 20, 40, 60, 90, 120, 150, and 180 minutes. Blood samples were taken from the rats tail vein using EDTA tubes. Blood samples were centrifuged at 3000 rpm for 15 minutes to obtain plasma, then stored at -80°C until analysis.

We used the previously validated LC-MS/MS method to determine curcumin concentration in biological matrices. The LC-MS/MS system used was UPLC Waters and tandem mass spectrometry with a positive electrospray ionization source. Separation of analyte in the sample was carried using a liquid chromatography system with Acquity UPLC^®^ BEH C18 column and mobile phase gradient in the form of 0.1% and acetonitrile formic acid with a ratio of 72:28 with analyte rate of 0.3 mL/min and injection volume of 3 µL. The retention time for curcumin and internal standards was in 2.5 and 1.6 minutes.^22,23^ Curcumin concentrations were calculated by extrapolation to the calibration curve from the standard curcumin solution. The calibration curve of a standard solution was made with the final level of the analytes at 1, 2.5, 5, 10, 25, and 50 ng/mL.^24^

The pharmacokinetic profiles were represented as mean and standard error (SE) from each group and were estimated through non-compartmental analysis. Pharmacokinetic parameters are calculated based on the curcumin concentration curve in plasma versus time. The area under the curve (AUC) from the beginning to the endpoint (${\text{AUC}}^{0\to 3}$) was calculated using the trapezoidal method. ${\text{AUC}}^{0\to \infty }$ is the amount of the AUC per time interval from 0 to the time of infinity, calculated by ${\text{AUC}}^{0\to 3}$ + Cpn; Cpn is the plasma concentration at the last sampling time. The terminal elimination rate constant (Ke) was estimated from the slope of the log-linear phase of a graph of the declining plasma concentration of curcumin versus time, and the absorption constant was calculated using the residual method. The t_1/2_ was counted using the equation t1/2 = ln 2/Ke, and the CL was calculated by dividing the dose by ${\text{AUC}}^{0\to \infty }$.^25^

### Statistical analysis

Data were presented as the mean ± SE. The student’s *t* test was used for assessing unequal variance. A *p* -value of less than 0.05 was considered significant. Statistical analyses were performed using SPSS (version 20, IBM, USA) software, and graphical representations were conducted using Microsoft Excel.

## Results

### Stability of curcumin nanoparticles

The stability of curcumin nanoparticles, such as color, turbidity, and sediment, were observed for five days after formulation. We found that there was no color change, turbidity was stable, and no curcumin deposits were found at the bottom of the vial.

### Particle size, PI and zeta potential

**Figure 1:**
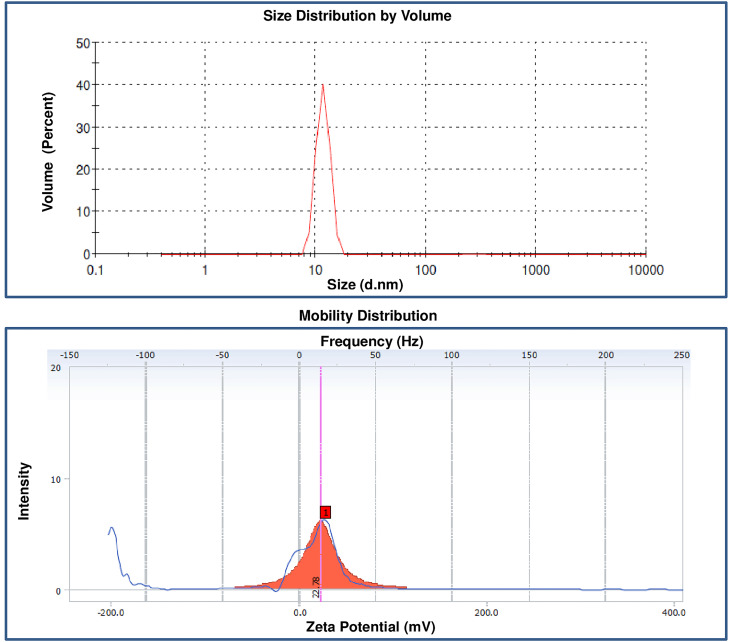
Particle size and zeta potential of curcumin nanoparticles.

The results of the particle size and zeta potential curcumin nanoparticle are given in Figure 1A-B. The mean particle size of the curcumin particle is 16103.4 nm with PI 0.847, and the curcumin nanoparticle is 11.5 nm with PI 0.509. The zeta potential of curcumin nanoparticle is +22.78.

### Examination of shape and morphology of curcumin nanoparticles

**Figure 2:**
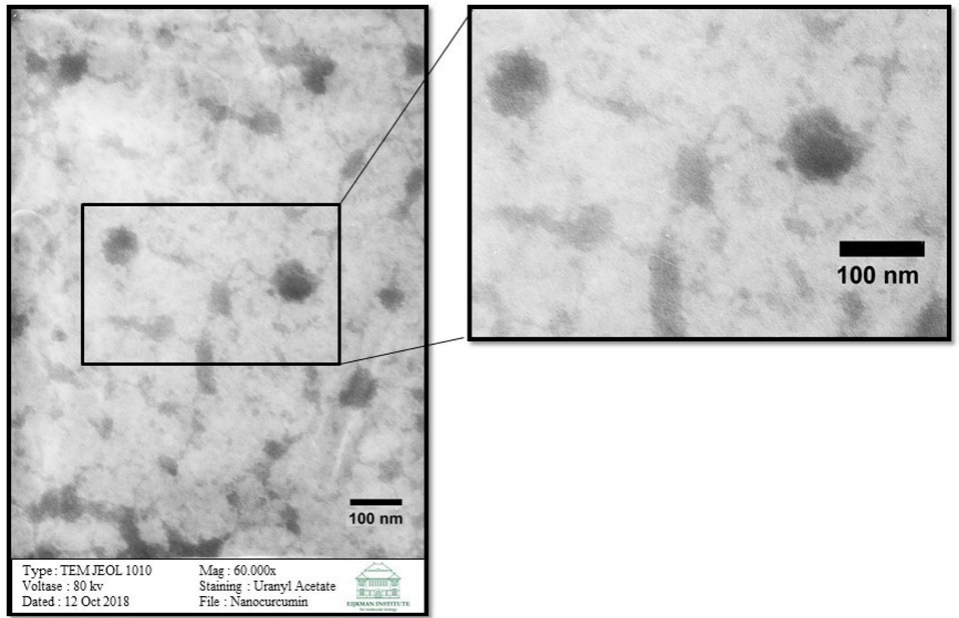
TEM image of curcumin nanoparticles.

The TEM examination results are shown in Figure 2. The TEM image of these curcumin nanoparticles is round.

### EE and DLC of the curcumin nanoparticles

The EE measurement of curcumin nanoparticles resulted in a relatively high yield, which was 99.97%. From this test, the average DLC was 11.34%.

### In vitro release of curcumin nanoparticles

**Figure 3:**
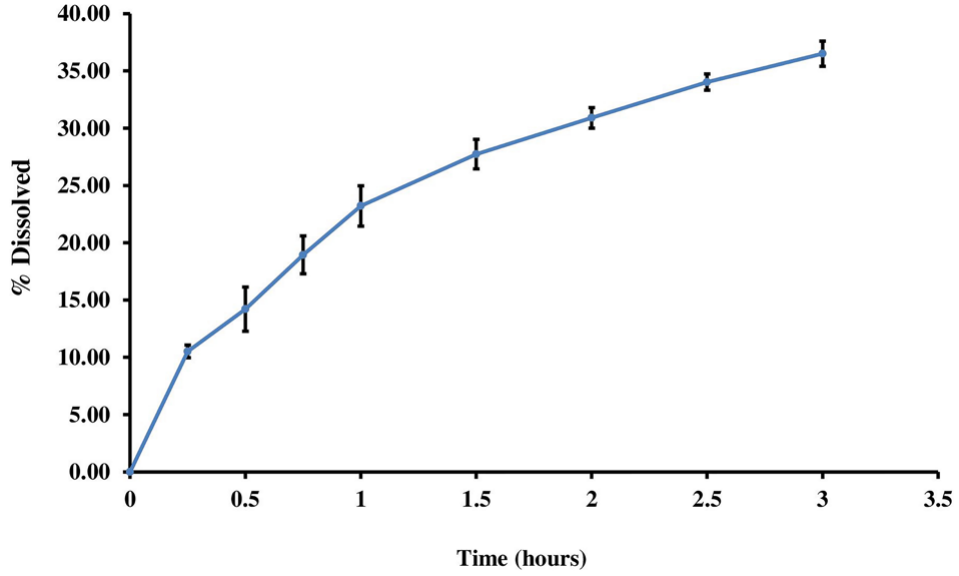
In vitro release profile of curcumin from curcumin nanoparticles in phosphate buffer medium.

*In vitro*, the release of curcumin nanoparticles was carried out to determine the release process and the amount of curcumin released from the curcumin nanoparticle matrix system. The result of the release test within 3 hours is given in Figure 3.

### Mucoadhesive properties of curcumin nanoparticles

**Figure 4:**
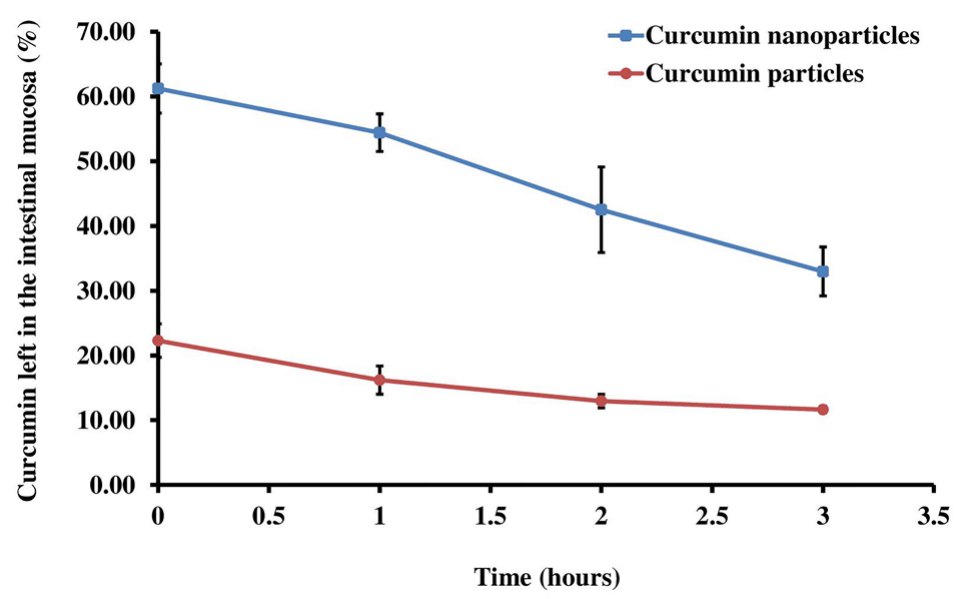
Mucoadhesive testing characteristics of curcumin and curcumin nanoparticles.

A mucoadhesive property test was conducted to determine the number of drugs that can attach to the intestinal mucosa during the time specified. There were differences of curcumin nanoparticles and curcumin particles on the amount of curcumin left in the intestinal mucosa (*P* < 0.05) (Figure 4).

### Pharmacokinetic profile of curcumin particles and curcumin nanoparticles in plasma after oral administration

In our study, measurements of plasma curcumin concentrations obtained maximum levels of 1.512 ng/mL for rats given curcumin particles and 16.585 ng/mL in groups given curcumin nanoparticles. The results of measuring the levels of curcumin particles or curcumin nanoparticles per milliliter of plasma showed that at various time intervals since the administration of the drug, the level of curcumin in the group that received curcumin nanoparticles in the plasma was higher than the group receiving curcumin particles. There are statistically significant differences (*P* < 0.05).

**Figure 5:**
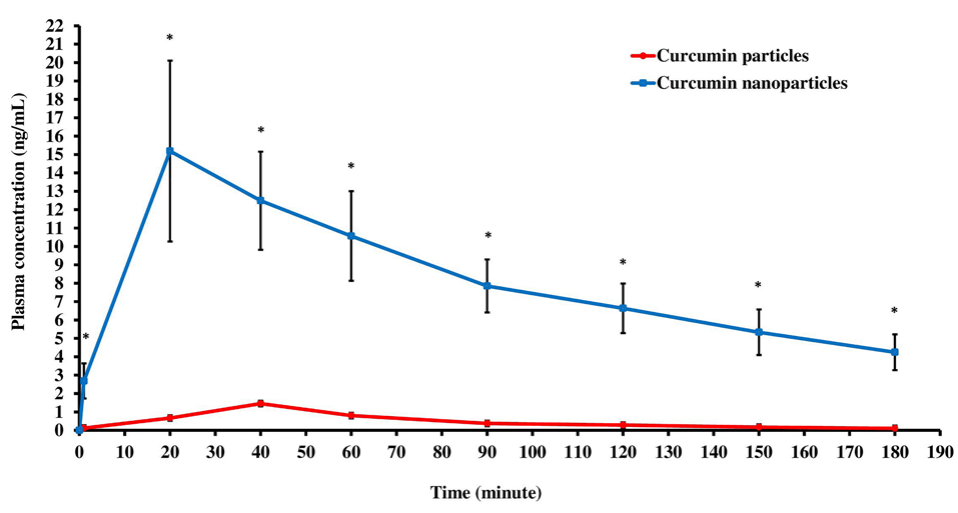
Plasma concentration-time curve of curcumin after oral administration of curcumin particles (100 mg/kg BW) and curcumin nanoparticles (100 mg/kg BW). The nanocurcumin group had a better pharmacokinetic profile than the curcumin group. These results were obtained 180 minutes after oral administration of drugs. * *P* <0.05 versus the curcumin group. *Each point represents the mean ± SE (n=5).

The results of plasma levels of curcumin particles and curcumin nanoparticles from 0 to 180 minutes (3 hours) after oral administration of drugs can be calculated. Pharmacokinetic parameters include AUC, maximum levels (Cmax), and time needed to reach maximum concentration (Tmax). The pharmacokinetic parameters in the curcumin nanoparticles group as a whole had a statistically significant difference (*P* < 0.05). Pharmacokinetic parameters are shown in Figure 5 and Table 1.

**Table 1:** Pharmacokinetic parameters of curcumin particles (100 mg/kg BW) and curcumin nanoparticles (100 mg/kg BW)

**Parameter(unit)**	**Curcumin particles**	**Curcumin nanoparticles**	**Ratio**	***P*** **value (<0.05)**
AUC_0-3(_ng.jam/mL)	1.498 ± 0.401	24.934 ± 4.924	1: 16.64	0.023
AUC_0-~(_ng.jam/mL)	1.713 ± 0.535	34.010 ± 7.016	1 : 19.85	0.009
Cmax (ng/mL)	1.512 ± 0.401	16.585 ± 3.818	1: 10.97	0.045
Tmax (min)	44 ± 3.5	24 ± 3.5	1 : 0.55	0.015
K_e_ (h^-1^)	1.178 ± 0.347	0.547 ± 0.068	1 : 0.46	0.120
t_1/2 elimination_(h)	0.859 ± 0.214	1.385 ± 0.201	1 : 1.61	0.585
K_a_ (h^-1^)	1.958 ± 0.468	4.514 ± 1.184	1 : 2.31	0.023
t_1/2 absorption_ (h)	0.523 ± 0.177	0.223 ± 0.057	1 : 0.43	0.185
Cl (L/kg/jam)	48.36 ± 14.76	1.74 ± 0.37	1 : 0.04	0.000

* Significantly different after student *t* test. Each point represents the mean ± SE (n = 5).

## Discussion

Our current formulation of curcumin nanoparticles resulted in satisfactory particle size, i.e., 11.55 nm. Our result shows that the curcumin nanoparticles prepared, met the requirements of having a particle size of 10–1000 nm, while unmodified curcumin has a particle size of 16 103.4 nm. The submicron-sized particles are useful for drug delivery models with controlled-release targets as carriers that mediate the drug model so that the drug directly works to the destination.^26,27^ The solubility of a substance or drug can be increased by formulating a drug and by reducing the particle size. Decreasing particle size will increase the area of particle surface so that interaction with solvents increases. Decreasing particle size below 1 µm will increase solvent pressure, which results in improved solubility and reduced interaction between solutes, which facilitates the dissolution process.^28^ In general, drugs that utilize nanoparticle formulation have low solubility in water and low oral bioavailability. Nanoparticles can increase the absorption of a compound so that the plasma concentrations increase, which was proven by the increase of AUC.

Curcumin has poor solubility in water and is lipophilic with a partition coefficient of 2.3–2.6.^7^ Curcumin is also easily degraded by light and at alkaline pH. Thus, the nanoparticle of curcumin using ionic gelation methods was prepared. This method is quite easy and straightforward. With this method, a large molecular weight complex is formed, which is dispersed into the form of similarly charged ions and is crossed with a lower molecular weight compound and in contrast to the polymer charge. To prepare a good formulation, curcumin must be appropriately dissolved to obtain a proper formulation. Curcumin is a nonpolar compound that is an unsaturated diketone compound, which has poor solubility in water. A large number of carbon clusters in curcumin make it difficult for water to dissolve.

The solubility of curcumin in water is very low, which is 400 ng/mL. Curcumin has a high solubility in DMSO and also soluble in ethanol. The use of DMSO and ethanol has a concentration limit in a formula, which is not allowed to exceed 10%.^29^ However, the use of cosolvent is not enough to increase the solubility of curcumin. Therefore, we added cosolvent, surfactants, and several other solvents, which can increase the solubility of curcumin. The addition of tween 80 as a stabilizing agent, which is a group of surfactants in curcumin, reduces the surface tension of curcumin so that the solubility of curcumin increases. After that, the mixed solvent used consisted of 70% ethanol, glycerin, and propylene glycol. DMSO 10% as a solubilizing agent is used to increase the solubility of curcumin. DMSO itself is a polar aprotic solvent that dissolves both in polar and nonpolar compounds. Kolliphor EL, polyoxyl 35 castor oil, is then used as a solvent from the reaction of castor oil with ethylene oxide so that it can increase the solubility of curcumin. With the addition of these solvents, curcumin can be completely dissolved. Then, chitosan and cross-linkers were added in the form of NaTPP with a drop rate of one drop/3 second until curcumin nanoparticles were formed with turbidity and color changes to yellow-orange.

In our experiment, curcumin nanoparticles at final formulation showed excellent stability up to 5 days after formulation. In the process of manufacturing nanoparticles using the ionic gelation method, this is influenced by the selection of polymers and cross-linkers. Chitosan is a polymer that has been commonly used and has many beneficial properties as polymeric materials. Chitosan is inert, biocompatible and has excellent mucoadhesive properties. Cross-linking agents in the form of NaTPP are the best cross-linker to prepare nanoparticles compared to other materials due to its many anions. The addition of NaTPP can correctly form a cross-link that makes ionic molecular interactions of chitosan ions with the addition of NaTPP anions. In our result, our curcumin nanoparticle index was 0.509, which fulfilled the particle size distribution requirements, thus indicating that curcumin nanosuspension was distributed homogeneously. The PI is a parameter that shows the homogeneity of a size of nanoparticle droplets and uniformity of particle size distribution. A good PI value is 0–1, where the closer to 0, the more uniform the distribution.^14,30^

From the measurement of curcumin nanoparticles, the *ζ* potential is +22.78. These results are quite far from the value of 0. They indicate the uniformity of positively charged particles in the suspension of nanoparticles made so that the possibility of aggregation between particles decreases, and the nanoparticle becomes stable. Zeta potential is a measure of the degree of repulsion between dispersed particles with the same charge and closes together. From this *ζ* potential measurement, we can see the magnitude of the electric charge between one particle and other particles in the nanoparticles, and this *ζ* potential value is related to the stability of the formed nanoparticles. A good *ζ* potential value is a value that keeps away from the number 0, both positive and negative. If the *ζ* potential value approaches 0, it can allow aggregation to occur, which makes an unstable nanosuspension. Different charges with the same amount of nanoparticles might lead to particle aggregation. Therefore, a higher charge will result in a more stable particle due to the higher resistance between particles.^27^ The positively charged nanoparticles will rapidly penetrate the mucus layer because they interact electrostatically with the negatively charged mucin.^31,32^ Rajendra et al showed that positively charged nanoparticles would be absorbed faster and eliminated longer (long t_1/2_ and small CL).^33^

From the TEM examination, in which this study obtained morphological results, it can be seen that the particles are round. The round form, which shows that curcumin nanoparticles are well-formed and will more easily enter the membrane. Of all the shapes, round nanoparticles are best suited for drug delivery applications.^14,30,34^

Curcumin nanoparticles are also used to determine the amount of curcumin that has been successfully absorbed in nanoparticles. The amount of curcumin absorbed can be determined by separating curcumin from the dispersing medium by centrifugation technique. The higher the absorption efficiency in nanoparticles, the better the nanoparticles that have been made. High absorption efficiency is very beneficial because it can transport enough drugs to the target organs and increase drug contact time.^35^ Our result showed that curcumin nanoparticles with chitosan polymer have succeeded in increasing adsorption efficiency. Drug-polymer interactions influence this absorption efficiency. From the results of this absorption efficiency, the ability of chitosan to bind medicinal ingredients is shown. The high adsorption efficiency was obtained because of the large protonated amine chitosan group. Thus, increasing the capacity of chitosan to bind curcumin that has a negative charge resulting in weak ionic interactions, which cause curcumin to be absorbed in the polymer matrix and produce high adsorption efficiency. The higher the concentration of chitosan in nanoparticles, the more amine groups that bind to curcumin, and the absorption efficiency is also high. NaTPP added is used as a cross-linker, by linking the remaining amine groups in one polymer that does not interact with curcumin. Hence, form strong ionic interactions with chitosan by having a negative charge. Consequently, creating stable nanoparticles and more curcumin can be absorbed in nanoparticles.

Another evaluation of nanoparticles is the determination of DLC. The results of this DLC affect the level of drug release from the carrier. Factors that influence the outcomes of DLC include the solubility of curcumin in the chemical bond chitosan matrix that occurs between curcumin and chitosan where the more robust the chemical bond, the more curcumin is absorbed in the nanoparticles and the higher the DLC.^35,36^

*In vitro* release profiles of curcumin from curcumin in the phosphate buffer showed a continuous increase in dissolved percentage in proportion to the time. From the curcumin concentration profiles, we presume that the release of nanoparticles might be a sustained-release since the release profile is not zero order patterns. The results obtained are the percentage of drugs that are released, which are still small within 3 hours and can continue to increase over time.^35^ The increased release tends to be continually starting from the first hour. These results match and resemble the results of the release of curcumin nanoparticles in other studies that obtained results as significant as 30%.^14^

The testing of mucoadhesive properties in this study was carried out to determine the number of drugs that can attach to the intestinal mucosa during the time specified. This test is essential to attach curcumin in the intestine and observe the effect of chitosan as a polymer used to improve the mucoadhesive properties of curcumin in nanoparticles.^37^ The mucoadhesive properties of curcumin nanoparticles showed superior attachment ability to the intestinal mucosa than curcumin. Improved mucoadhesive characteristics of curcumin nanoparticle, are due to its higher solubility and well-mixed with chitosan. Chitosan is known to have excellent mucoadhesive properties. Chitosan, which has a positive charge, will ionically bind with a negative charge from mucus, resulting in the formation of a strong bond is formed. Thus, allowing curcumin to penetrate in a longer time. With an excellent mucoadhesive property, curcumin nanoparticle will result in increased penetration and longer intestinal residence time. Thus, increased absorption and bioavailability will be achieved. *In vivo* pharmacokinetic study last for 3 hours to measure curcumin plasma concentrations. Our result showed a significant improvement in overall pharmacokinetic profiles. We demonstrated a substantial increase of AUC and Tmax of curcumin nanoparticles as compared to curcumin particles. In the early times of sampling, which was the absorption phase, curcumin nanoparticles are absorbed faster than curcumin particles and reach Tmax within 24 minutes, while curcumin particles within 44 minutes. The result indicates that decreasing particle size can improve the absorption process. Another study states that the Tmax obtained from the curcumin nanoparticles was 30–90 minutes.^16^ The data shows that curcumin nanoparticles in this study reached faster Tmax. Absorption is one of the critical stages of oral drug administration. Drug permeability in membranes, barrier mucus on the surface of enterocytes, and efflux transporters are factors that influence absorption in the gastrointestinal tract. Decreasing particle size is expected to improve the absorption process because it can increase the solubility of a compound. Nanosize particles accelerate the translocation process in mucus and further cross the gastrointestinal membrane through transcellular transport, such as endocytosis mediated by caveolar and clathrin or pinocytosis.^31,38^ Particle translocation kinetic in mucus depends on diffusion and access through mucus. The speed of particles diffusing across mucus is influenced by several factors, such as particle size and charge. The smaller the particle size, the faster it crosses the digestive tract.

The study conducted by Timothy et al showed that maximum concentrations of curcumin were 6.5 nM with Tmax of 30 minutes at doses of 340 mg/kg given orally, while Maiti obtained a Cmax of 0.5 mg/mL at 45 minutes using a dose of 1 g/kg BW orally.^39^ The difference in Cmax of curcumin suspension was also seen in liposome preparation of curcumin nanoparticles as large as 71,35 ng/L.^40^ In the PLGA study, Cmax of the curcumin suspension in the curcumin nanoparticles was as large as 4 ng/mL^41^ at the same dose (50 mg/kg BW orally). Curcumin nanoparticles encapsulated with liposomes showed an increase in Cmax twice higher than curcumin suspension.^40^ Cmax increase was also seen in curcumin nanoparticles encapsulated with PLGA 3 times and with PLGA-PEG 7.5 times compared to curcumin suspension. The effect of particle size on the rise in the absorption process is where plasma levels are seen to be higher in the study of encapsulated curcumin nanoparticles.^41^

The AUC value in this study appeared to be statistically significantly different between the two groups. The AUC in the curcumin nanoparticles group was 16 times greater than the group that received curcumin particles. The role of decreasing particle size in increasing the amount of drug in the blood is seen in this study. Our result shows the similarity with other curcumin nanoparticle research conducted in several studies. Research conducted by Shaikh et al showed an increase in the AUC value of 10 times when curcumin was encapsulated with PLGA polymers as nanoparticles,^42^ and also Najeh et al showed an increase in the AUC value of more than 15.6 and 55.4 times when curcumin was encapsulated with PLGA, and PLGA-PEG polymers served as nanoparticles.^41^ In general, nanoparticle (nanoformulation) studies, the amount of drug in the body (AUC) will increase along with increased t_1/2_ and decreased CL. Nanoparticles with polymers can avoid the RES so that they are slightly metabolized and eliminated from the body. Thus, the drug will be circulated for a longer time, which is marked by increased t_1/2_ and decreased CL.^41^ In this study, there were increased in AUC, which was accompanied by longer t_1/2_ and lower CL in the curcumin nanoparticle group vs. unmodified curcumin. Improvement in nanocurcumin pharmacokinetic profiles can be explained by the encapsulation of curcumin molecules, which offers protection from rapid degradation in the body. Other pharmacokinetic parameters such as t_1/2_ and measured CL in plasma in this study showed a statistically significant difference where the t_1/2_ in the curcumin nanoparticle group was longer, and the CL was smaller.

## Conclusion

We conclude that a cost-effective and straightforward nanoparticle formulation of curcumin based on chitosan-NaTPP can be conducted to achieve a particle size of 30 nm, *ζ* potential of 22.78 mV, PI of 0.509, EE of more than 99%, and DLC of 11%. The above formulation resulted in a significant increase in mucoadhesive properties and improvement in pharmacokinetic profiles of curcumin nanoparticles. Therefore, chitosan-NaTPP-based curcumin nanoparticles are a promising candidate for further use in various diseases.

## Ethical Issues

The pharmacokinetic evaluation was carried out after obtaining approval from the Ethics Committee of the Faculty of Medicine Universitas Indonesia (approval number: 1188/ UN.2 F1/ETIK/2018).

## Conflict of Interest

The authors declare no conflict of interest.

## Acknowledgments

This study was funded by the Indonesian Ministry of Research Technology and Higher Education through World-Class Research (WCR) 2019 grant. We thanked Enago Academic Editing Services (https://www.enago.com) for providing English language editing on this paper..
